# Threshold Values of Myopic Anisometropia Causing Loss of Stereopsis

**DOI:** 10.1155/2019/2654170

**Published:** 2019-05-06

**Authors:** Maciej Gawęcki

**Affiliations:** Dobry Wzrok Ophthalmological Clinic, Kliniczna 1B/2, 80-402 Gdansk, Poland

## Abstract

**Purpose:**

The aim of the study was to determine the threshold values of myopic anisometropia that lead to the loss of stereoacuity in most of patients.

**Materials and Methods:**

Forty healthy subjects were included in the study. The inclusion criteria were as follows: lack of any functional or morphological ophthalmological disorders, or detectable damage to the visual system, anisometropia equal or less than 0.25 D in a spherical equivalent, and full stereoscopic vision for near and for distance. Myopic anisometropia was evoked by placing different focusing lenses in front of the right eye of the subject in the trial frame. Stereoscopic vision was assessed with the use of the Titmus test (dots) (Stereo Fly Test Stereo Optical Co. Inc.) for near and the Randot test for distance (Distance Randot Stereotest Stereo Optical Co. Inc.).

**Results:**

The threshold values for different types of myopic anisometropia for the loss of stereopsis in more than 50% of patients were determined. For near, this value was 3 D for sphere and “against the rule astigmatism” and 4 D for “with the rule astigmatism”. For distance, the values were 2 D for sphere and “against the rule astigmatism” and 3 D for “with the rule astigmatism.” *Conclusions*. Myopic anisometropia of more than 2 D can cause a significant impairment of binocular vision. Stereoacuity at distance is more sensitive to myopic anisometropia than stereoacuity at near. Myopic anisometropia involving “against the rule” astigmatism potentially affects binocularity more than anisometropia with regular astigmatism. A prompt correction of anisometropia of more than 2 D is needed in children to prevent the development of amblyopia.

## 1. Introduction

Anisometropia is a well-known risk factor for the development of amblyopia and sometimes strabismus. If significant and not corrected in the first years of life, it can disturb the normal development of the visual system. Visual acuity in the eye with a larger refraction error is usually decreased, and image in that eye is defocused. This leads to the asymmetry of the signals emerging from both eyes and the underdevelopment of the neurons driven by the defocused image on the level of the brain [1, 2]. Hypermetropic anisometropia is thought to be a more significant risk factor for the development of amblyopia than myopic anisometropia [3]. It can lead to fixation instability and mimic microstrabismus [4]. Myopic anisometropia is often treated as a benign form of anisometropia, which can be successfully treated even in older children. However, relatively little is known about its negative influence on the development of stereopsis. Stereoscopic vision is one of the most important properties of the visual system, which determines the quality of life and has an impact on the future professional career. Deficits in stereoscopic vision affect precision movements, precision grasping, and sense of distance [5]. Therefore, a lack of stereoscopic vision can limit personal engagement in professional life and hence causes frustration or even depression [6, 7]. Impaired stereoscopic vision is one of the most important deficits associated with anisometropic amblyopia [8]. The relationship between the amount of anisometropia and the loss of stereoacuity is yet to still be discussed in the medical literature. Controversies refer to the number of dioptres of anisometropia and the type of anisometropia (myopic, hyperopic, or astigmatic) that are the most likely to cause abnormalities in the visual system. Most of the studies that analyse the relationship between stereoscopic vision and the amount of anisometropia are population based—they study patients that are anisometropic and often amblyopic already [9]. As we know, the refraction error can change during the first years of life, so measurements that are taken in a few-year-old patient do not necessary reflect the maximum amount of anisometropia that was previously present in a subject, hence the idea of measuring stereopsis in healthy subjects after experimentally induced myopic anisometropia. Our study sought to determine the threshold amounts of myopic anisometropia for sphere and cylinder, which cause a loss of binocularity for near and for distance in healthy young individuals.

## 2. Materials and Methods

The study was conducted on 40 healthy subjects with no visual problems: 21 females and 19 males. The mean age of the patients was 34.9 ± 11.26 years. The inclusion criteria were as follows: lack of any functional or morphological ophthalmological disorders or detectable damage to the visual system, anisometropia equal or less than 0.25 D in spherical equivalent (SE), and full stereoscopic vision for distance and for near.

All subjects have undergone a routine ophthalmological examination that included the assessment of best-corrected visual acuity (BCVA) for distance and for near, slit lamp examination of the anterior segment of the eye, and indirect fundus examination by plus 90 D lens.

The refraction error was measured after cycloplegia with topical 1% tropicamide. Drops were administered twice with an interval of 5 minutes, and refraction was than measured after 40 minutes with an Oculus Park 1 autorefractometer (OCULUS, Germany 2008). The result was converted to SE, and the amount of anisometropia was than calculated. All subjects with anisometropia of more than 0.25 D were excluded from the study.

BCVA and stereopsis were measured on another day than the refraction error was determined. BCVA was measured on the Snellen chart. None of the patients required a distance optical correction. All subjects had full-distance visual acuity (BCVA) without correction: 1.0 Snellen. Some patients required a simple spherical optical correction for near, which was determined after the presence of anisometropia was excluded. Patients who did not achieve full near visual acuity after optical correction were excluded from the study.

Stereoscopic vision was assessed with the use of the Titmus test (dots) (Stereo Fly Test Stereo Optical CO Inc) for near and the Randot test for distance (Distance Randot Stereotest Stereo Optical Co Inc). The Titmus test with dots was suitable for adults as it provides precise grading of values of stereopsis for near expressed in seconds of arch. Unfortunately, there are not many distance stereotests available on the market. Randot stereotest for distance is one of the few officially approved for such testing, so it became our choice in current research. However, it has to be taken into consideration that distance Randot stereotest provides only 4 values of grading of stereoacuity. Only patients with full stereoscopic vision for distance and for near after optical correction were included in the study. Full stereoscopic vision was considered 40 sec of arch for near and 60 sec of arch for distance, as these were the minimal arch values measured on the abovementioned tests.

Myopic anisometropia was evoked by placing focusing lenses in front of the right eye of the subject in the trial frame. First in order, spherical focusing lenses were placed in the trial frame. Stereotest for distance and for near was than performed for +1 D, +2 D, +3 D, and +4 D powers of the lens. The same procedure was conducted for cylindrical lenses for + 1 D, +2 D, +3 D, and +4 D values. The cylinder was placed first in a 90-degree position (evoking “with the rule” astigmatism) and then in a 180-degree position (evoking “against the rule” astigmatism). Both stereotests were performed for each position of the cylinder lens.

As stereotests do not measure the amount of stereopsis in a linear way; therefore, grading of the results was established according to the achieved angle of stereopsis in the test.

Grading of stereoscopic vision is presented in Table 1.

The percentage of patients with different levels of stereopsis was then referred to each amount of anisometropia. The study sought the threshold amount of anisometropia that caused a loss of stereopsis in more than 50% of subjects.

## 3. Statistical Analysis

The statistical analysis was conducted with Statistica 13.0 software (StatSoft Inc., 2011). For the verification of statistical hypothesis, the ANOVA test of Friedman rank was used, including a post hoc test. The level of confidence was set at 0.05. The results were considered statistically significant if the calculated test probability was <0.05.

## 4. Results

The results for the loss of stereopsis at near for different forms of myopic anisometropia are presented below.

**Figure 1:**
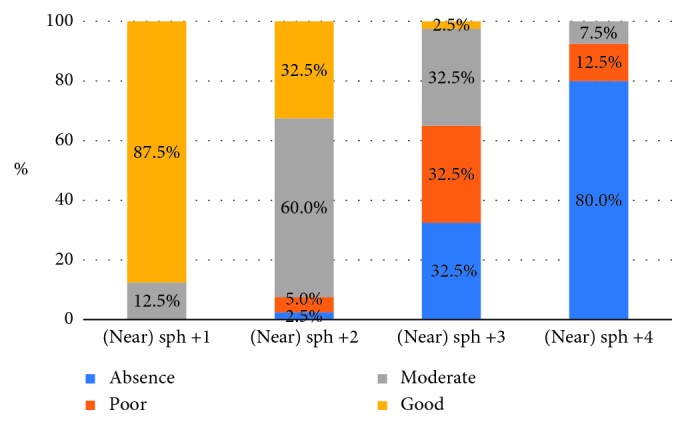
Distribution of patients with different grades of stereopsis according to the level of spherical myopic anisometropia presented on the graph.

The distribution of patients losing stereopsis with an increasing amount of spherical anisometropia for near is presented in Table 2 and Figure 1. The results of the post hoc ANOVA test are presented in Table 3.

Result of *χ*^2^ ANOVA (*N*=40, df = 3) = 108.0763, *p*=0.00000, so an increasing amount of spherical myopic anisometropia impairs stereopsis at near.

As we see in Table 3, there are significant differences for sph +1.0 D versus sph +3.0 D and sph +4.0 D and sph +2.0 D versus + sph +3.0 D and sph +4.0 D.

Most of the patients lose stereopsis when spherical myopic anisometropia equals 4.0 D; however, as the measurements of stereopsis show no statistical difference between +3.0 and +4.0 D, we can assume that 3.0 D is a threshold value of spherical myopic anisometropia for the loss of stereopsis at near.

**Figure 2:**
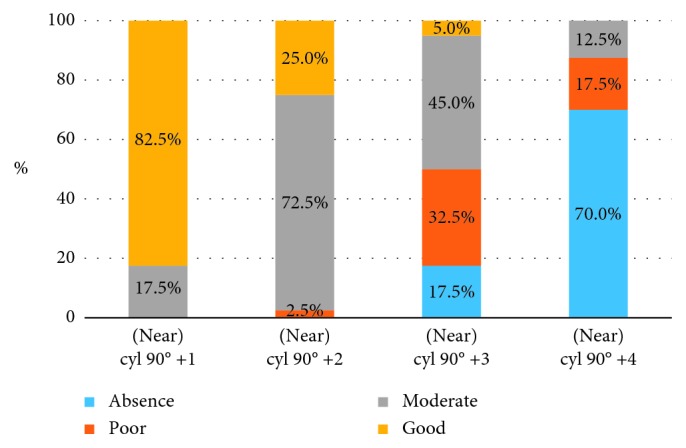
Distribution of patients with different grades of stereopsis for near according to the level of astigmatic myopic anisometropia (“with the rule” astigmatism) presented on the graph.

The results for the loss of stereopsis at near in astigmatic myopic anisometropia (“with the rule” astigmatism) are presented in Tables 4 and 5 and Figure 2.

Results of *χ*^2^ ANOVA (*N*=40, df = 3) = 105.7009 *p*=0.00000. An increasing amount of “with the rule” astigmatism significantly impairs stereopsis.

As we see from Table 5, the difference in measurements between cyl 90° +1.0 and cyl 90° +2.0 is not significant. All other pairs of measurements show a statistical difference.

Most of the patients lose stereopsis when myopic astigmatic anisometropia for “with the rule” astigmatism is 4 D. +3 D cyl 90° value also significantly impairs stereopsis; however, most of the patients in this group preserve some degree of binocularity.

**Figure 3:**
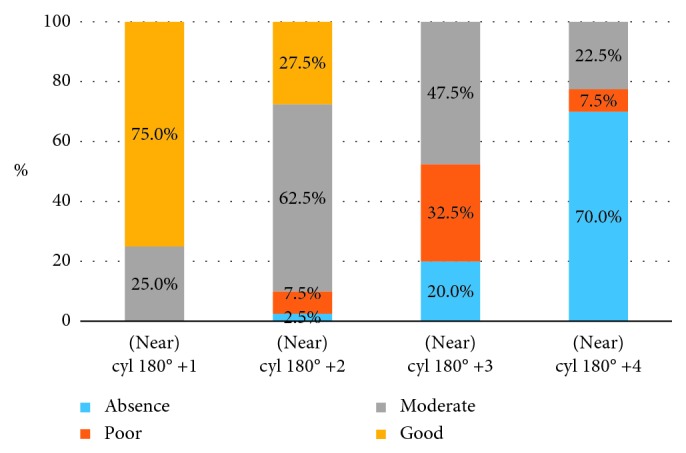
Distribution of patients with different grades of stereopsis according to the level of astigmatic myopic anisometropia (“against the rule” astigmatism) presented on the graph.

Analogous results for the measurements of stereopsis in myopic astigmatic anisometropia (“against the rule” astigmatism) are presented in Tables 6 and 7 and Figure 3.

Results of *χ*^2^ ANOVA (*N*=40, df = 3) = 101.3104 *p*=0.00000. An increasing amount of astigmatic anisometropia (“against the rule” astigmatism) significantly impairs stereopsis.

As we see from Table 7, there is no statistical difference for the stereopsis loss between +3.0 D cyl 180° and +4.0 D cyl 180°, so a value of 3 D of astigmatism, in this case, has to be treated as the threshold value for the loss of stereoacuity.

The results for the loss of stereopsis at distance for different forms of myopic anisometropia are presented below.

**Figure 4:**
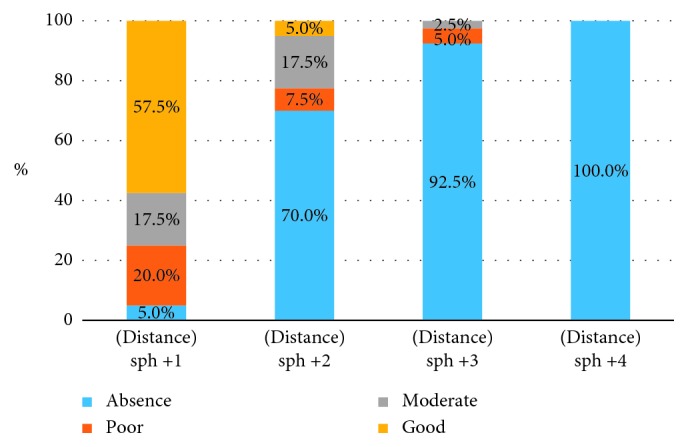
Distribution of patients with different grades of stereopsis at distance according to the level of spherical myopic anisometropia presented on the graph.

Tables 8 and 9 and Figure 4 present the impairment of stereopsis in spherical myopic anisometropia at distance.

**Figure 5:**
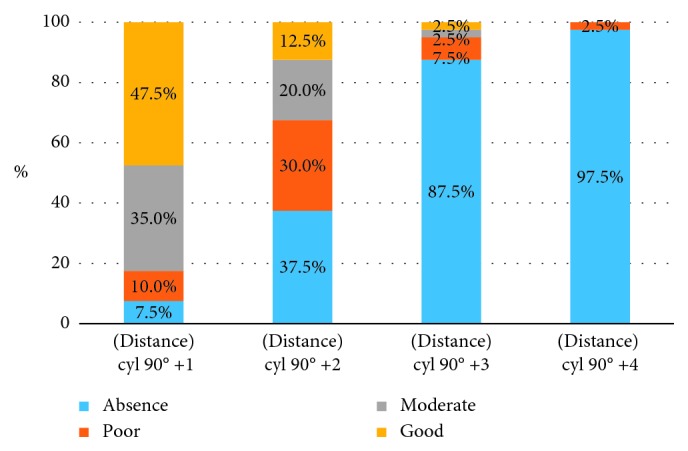
Distribution of patients with different grades of stereopsis for distance according to the level of astigmatic myopic anisometropia (“with the rule” astigmatism) presented on the graph.

Tables 10 and 11 and Figure 5 show the results of the change of stereoacuity with an increasing amount of myopic astigmatic anisometropia (“with the rule” astigmatism) at distance.

Results of *χ*^2^ ANOVA (*N*=40, df = 3) = 98.20152 *p*=0.00000. An increasing amount of spherical myopic anisometropia impairs stereopsis at distance.

As we see from Table 9 and Figure 4, as low as 2 D of myopic spherical anisometropia causes a loss of stereopsis at distance in most of the subjects.

Results of *χ*^2^ ANOVA (*N*=40, df = 3) = 90.07807 *p*=0.00000. An increasing amount of “with the rule” astigmatism impairs grade of stereoacuity.

As can be seen, most of the subjects lose stereoacuity at the level of anisometropia of 3 D for “with the rule” myopic astigmatism; however, a value of 2 D also significantly reduces the level of binocularity.

**Figure 6:**
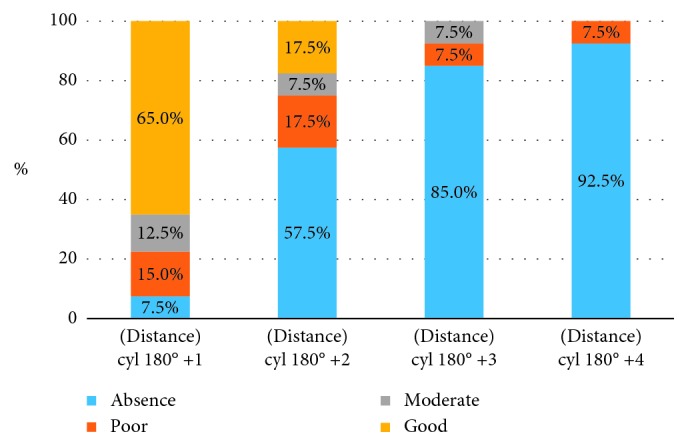
Distribution of patients with different grades of stereopsis at distance according to the level of astigmatic myopic anisometropia (“against the rule” astigmatism) presented on the graph.

Tables 12 and 13 and Figure 6 present the results for the measurements of stereopsis at distance in myopic anisometropia involving “against the rule” astigmatism.

Results of *χ*^2^ ANOVA (*N*=40, df = 3) = 90.07807 *p*=0.00000. An increasing amount of “against the rule” astigmatism impairs grade of stereoacuity.

As can be seen from the above data, 2 D of myopic anisometropia with “against the rule” astigmatism leads to a loss of binocularity in most patients.

A summary of the threshold values of myopic anisometropia causing a loss of stereopsis is presented in Table 14.

## 5. Discussion

In population-based studies, anisometropia is indicated as an important factor affecting stereoacuity although there are controversies regarding threshold values for its loss. Levi et al. analysed 84 pure anisometropes according to the loss of stereopsis. Myopic anisometropes showed much better stereopsis than analogues anisohypermetropes. In pure anisometropia, there was a linear relationship between the increasing amount of anisometropia and the loss of stereopsis [9]. Dobson et al. depicted a population of school-aged children with a high prevalence of astigmatism [10]. In this study, a significant increase in the presence of amblyopia referred only to hyperopic anisometropia of 1 D or more in sphere or 2-3 D or more in astigmatism. However, a significant reduction of stereoacuity was noted in anisometropia as low as 0.5 D or more in sphere or cylinder for all refraction errors. Jeon and Choi analysed 107 children with anisometropia [11]. The children were divided into 2 groups: amblyopic and nonamblyopic. The average degree of anisometropia was 2.54 in the nonamblyopic group and 4.29 D in the amblyopic group. Stereopsis was significantly worse in the amblyopic group: 641.71 sec. of arch versus 76.25 sec. of arch., while it was 54.52 sec. arch in the controls. In the study by Chen et al., pure anisometropes of 3 D or less retain fusion and some stereopsis. A complete loss of binocularity was noted in anisometropia as high as 6 D or more [12]. Yan et al. report an impairment of stereopsis in children with myopic anisometropia of more than 1 D in sphere or cylinder [13].

As can be reasoned from the abovementioned studies, anisometropia affects stereoacuity, but it is difficult to name the amount of anisometropia that significantly reduces stereoscopic vision. In population-based studies, researchers often deal with stereoacuity defects of different origins (anisometropia, microstrabismus, and deprivation), which makes such an analysis difficult.

On the contrary, studies analysing stereopsis in experimentally induced anisometropia enable to precisely measure the deficiency of stereoacuity per 1 D of ametropia.

Oguz and Oguv experimentally induced anisometropia in healthy adults [14]. In this study, stereoacuity was reduced by 57–59 sec. of arch for 1 D of spherical anisometropia and 51–56 sec. of arch for astigmatism. The threshold value of anisometropia, which significantly reduced stereoacuity, was 3 D for both sphere and cylinder. Similar results were reported by Dadeya et al. and Gawęcki and Adamski [15, 16]. Kulkarni et al. analysed the influence of experimentally induced astigmatism on stereoacuity [17]. The authors used 2 values of astigmatism: 1 D or 2 D placed on a different axis. The stereoacuity levels decreased with the increase of the dioptre power of astigmatism. They were affected the most by the oblique astigmatism and the least by the astigmatism at the 180 axis. A similar study for astigmatism was performed by Al-Qahtani, and Al-Debasi confirmed these results [18].

The present study analyses myopic anisometropia in particular. In comparison to previous reports, it employs grading of stereopsis and is performed on a relatively large number of patients. In this research, the threshold values of myopic anisometropia, which lead to a complete loss of stereoacuity, differ in near and distance measurements. Myopic anisometropia is better tolerated for near, where values of 3-4 D cause a loss of binocularity. At distance, as low as 2 D of anisometropia can significantly decrease or cause a loss of binocularity. We also observe that “against the rule” astigmatism can affect stereoacuity more than regular astigmatism. These results are in consent with previous studies; however, this paper additionally presents intermediate values of anisometropia that impair stereoacuity, but not suppress it totally. It has to be remembered that lower threshold values of myopic anisometropia also put patients at risk of developing amblyopia.

Determining the threshold values for the loss of stereoacuity has practical therapeutic implications. Diagnosing a child with myopic anisometropia of 2 D or more implicates the need for immediate treatment. Therapeutic decisions have to be determined by the presence of the sensitive period for the treatment of amblyopia, available therapeutic methods, and potential risks associated with the application of those methods.

Most of the studies indicate the sensitive period for visual development as age 0–7 [19–21]. However, there is evidence that supports more effective treatment of amblyopia in younger children [22, 23]. Donahue reports a low prevalence of amblyopia in anisometropic children aged less than 3 [24]. After the age of 3, in most children with anisometropia, amblyopia is already developed.

Correction of the refraction error including anisometropia is a key for preserving and restoring binocularity. Without such treatment chances for normal development of the visual system are significantly diminished. A smaller amount of anisometropia can be successfully corrected with glasses, and a larger amount with contact lenses [25]. However, there exist a number of children uncompliant to optical correction by those means. In such cases, laser correction of the refraction error should be considered. Medical literature presents successful functional results of PRK in anisometropic children. Autrata et al. reports good binocular function in 13 children aged 7–15 who underwent photorefractive keratectomy (PRK) in high myopic anisometropia [26]. Twelve of the thirteen patients had a fusional potential, and 6 of them had stereopsis. In a later study, the same authors present a better binocular function in anisometropic children after PRK or laser-assisted subepithelial keratectomy (LASIK) than in anisometropic children treated by contact lenses (fusion and stereopsis gain in 78% versus 33%) [27]. Paysee et al. also report optimistic results in anisometropia treated by PRK [28, 29]. Stereopsis improved in 33% of cases (short term) and 55% of cases (long term) of children between 2 and 11 years of age. Yin et al. analysed 32 myopic children who underwent LASIK due to myopic anisometropia [30]. The number of patients who had stereopsis improved from 19% before to 89% after the surgery. Astle et al. reported the percentages of stereopsis gain from 39.4% to 87.9% for the whole cohort of children with hyperopic and myopic anisometropia [31]. The improvement of stereopsis in anisometropic patients after corneal refractive surgery also applies to adults [32].

Magli et al. published less optimistic results [33]. Just 2 of 18 patients with myopic anisometropia improved stereopsis after PRK. Similar results were reported by Zangh and Yu in juvenile patients with myopic anisometropic amblyopia, who had no stereopsis before femtosecond laser corneal surgery [34]. There was a stereopsis gain in 21.2% of these patients.

The other method of correcting large anisometropia is phakic intraocular lens (p-IOL) implantation. The procedure involves the implantation of an artificial lens either into the anterior chamber or into the ciliary sulcus with a preservation of the natural lens of the patient. Tian et al. performed a meta-analysis of the literature on the subject [35]. They compared the functional improvement of vision in children with myopic anisometropia after corneal refractive surgery and after p-IOL implantation. Binocular vision improved in more than half of the patients in both groups.

Just recently, implantable collamer lenses (ICL) have been introduced for the correction of large refractive errors. They are p-IOLs implanted to the ciliary sulcus. Zhang et al. report a treatment of 11 eyes of children with unilateral high myopia (average age of 11 years) treated with ICL. The procedure resulted in a significant improvement of BCVA; however, none of the patients had a stereopsis recovery for near after the surgery [36]. The same author reports the effects of ICL treatment in adults with myopic anisometropia [37]. A basic stereopsis gain for near was noted in 4 of 13 patient who underwent the procedure.

As we see from the listed studies, the results of surgical treatment are satisfactory just in some cases. This may be due to the age of patients that undergo the surgery, which is usually advanced as for the amblyopia treatment. Like in every therapy, the potential risks of such a surgery have to be balanced with the potential benefits. Phakic IOLs, especially ICLs, seem to be reasonable treatment options for children with large anisometropia in whom correction with contact lenses or glasses is impossible or troublesome. This applies especially to high myopic anisometropia, as high myopia is often difficult to be corrected by corneal laser surgery. Besides, myopia is a refraction error that is willingly corrected by many patients when they reach adult age. In the case of myopic anisometropia, a decision about the surgery should be undertaken within the sensitive period for visual development.

## 6. Conclusion

Myopic anisometropia of more than 3 D in sphere or cylinder causes a total loss of stereopsis for near in most patients. At distance, myopic anisometropia as low as 2 D results in a significant impairment or loss of binocularity. Myopic anisometropia involving “against the rule” astigmatism disturbs stereoacuity more than anisometropia involving “with the rule” astigmatism. Immediate measures for optical or sometimes surgical correction should be undertaken if myopic anisometropia of 2 D or more is diagnosed during screening for the refraction error in children. There is a need for creating an algorithm for the treatment of anisometropic amblyopia that would consider the age of patients, their compliance, and the amount of anisometropia.

## Figures and Tables

**Table 1:** Classification of the degree of stereopsis.

Grade of stereopsis	Near (sec. of arch)	Distance (sec. of arch)
Good	40, 50, 60, 80, 100	60, 100
Moderate	140, 200, 400	200
Poor	800	400
Absence	∝	∝

**Table 2:** Distribution of patients with different degrees of stereopsis for near according to the amount of spherical anisometropia.

Grade of stereopsis	(Near) sph +1		(Near) sph +2		(Near) sph +3		(Near) sph +4	
	*n*	%	*n*	%	*n*	%	*n*	%
Absence	0	0.0	1	2.5	13	32.5	32	80.0
Poor	0	0.0	2	5.0	13	32.5	5	12.5
Moderate	5	12.5	24	60.0	13	32.5	3	7.5
Good	35	87.5	13	32.5	1	2.5	0	0.0
Total	40	100.0	40	100.0	40	100.0	40	100.0

**Table 3:** Results of the post hoc ANOVA test showing the statistical difference between all possible pairs of measurements. Absolute differences between mean rank values are significant if larger than 0.761599273516645 at a confidence level = 0.05.

	(Near) sph +1	(Near) sph +2	(Near) sph +3	(Near) sph +4
(Near) sph +1	—	0.6375	1.875	2.5375
(Near) sph +2	0.6375	—	1.2375	1.9
(Near) sph +3	1.875	1.2375	—	0.6625
(Near) sph +4	2.5375	1.9	0.6625	—

**Table 4:** Distribution of patients with different degrees of stereopsis for near according to the amount of astigmatic anisometropia (“with the rule” astigmatism).

Grade of stereopsis	(Near) cyl 90° +1		(Near) cyl 90° +2		(Near) cyl 90° +3		(Near) cyl 90° +4	
	*n*	%	*n*	%	*n*	%	*n*	%
Absence	0	0.0	0	0.0	7	17.5	28	70.0
Poor	0	0.0	1	2.5	13	32.5	7	17.5
Moderate	7	17.5	29	72.5	18	45.0	5	12.5
Good	33	82.5	10	25.0	2	5.0	0	0.0
Total	40	100.0	40	100.0	40	100.0	40	100.0

**Table 5:** Results of the post hoc ANOVA test showing the statistical difference between all possible pairs of measurements. Absolute differences between mean rank values are significant if larger than 0.761599273516645 at a confidence level = 0.05.

	(Near) cyl 90° +1	(Near) cyl 90° +2	(Near) cyl 90° +3	(Near) cyl 90° +4
(Near) cyl 90° +1	—	0.725	1.6625	2.6125
(Near) cyl 90° +2	0.725	—	0.9375	1.8875
(Near) cyl 90° +3	1.6625	0.9375	—	0.95
(Near) cyl 90° +4	2.6125	1.8875	0.95	—

**Table 6:** Distribution of patients with different degrees of stereopsis for near according to the amount of astigmatic anisometropia (“against the rule” astigmatism).

Grade of stereopsis	(Near) cyl 180° +1		(Near) cyl 180° +2		(Near) cyl 180° +3		(Near) cyl 180° +4	
	*n*	%	*n*	%	*n*	%	*n*	%
Absence	0	0.0	1	2.5	8	20.0	28	70.0
Poor	0	0.0	3	7.5	13	32.5	3	7.5
Moderate	10	25.0	25	62.5	19	47.5	9	22.5
Good	30	75.0	11	27.5	0	0.0	0	0.0
Total	40	100.0	40	100.0	40	100.0	40	100.0

**Table 7:** Results of the post hoc ANOVA test showing a statistical difference between all possible pairs of measurements. Absolute differences between mean rank values are significant if larger than 0.761599273516645 at a confidence level = 0.05.

	(Near) cyl 180° +1	(Near) cyl 180° +2	(Near) cyl 180° +3	(Near) cyl 180° +4
(Near) cyl 180° +1	—	0.7	1.7625	2.4375
(Near) cyl 180° +2	0.7	—	1.0625	1.7375
(Near) cyl 180° +3	1.7625	1.0625	—	0.675
(Near) cyl 180° +4	2.4375	1.7375	0.675	—

**Table 8:** Distribution of patients with different degrees of stereopsis for distance according to the amount of spherical anisometropia.

Grade of stereopsis	(Distance) sf +1		(Distance) sf +2		(Distance) sf +3		(Distance) sf +4	
	*n*	%	*n*	%	*n*	%	*n*	%
Absence	2	5.0	28	70.0	37	92.5	40	100.0
Poor	8	20.0	3	7.5	2	5.0	0	0.0
Moderate	7	17.5	7	17.5	1	2.5	0	0.0
Good	23	57.5	2	5.0	0	0.0	0	0.0
Total	40	100.0	40	100.0	40	100.0	40	100.0

**Table 9:** Results of the post hoc ANOVA test showing a statistical difference between all possible pairs of measurements. Absolute differences between mean rank values are significant if larger than 0.761599273516645 at a confidence level = 0.05.

	(Distance) sf +1	(Distance) sf +2	(Distance) sf +3	(Distance) sf +4
(Distance) sf +1	—	1.5	1.9625	2.0375
(Distance) sf +2	1.5	—	0.4625	0.5375
(Distance) sf +3	1.9625	0.4625	—	0.075
(Distance) sf +4	2.0375	0.5375	0.075	—

**Table 10:** Distribution of patients with different degrees of stereopsis for near according to the amount of astigmatic anisometropia (“with the rule” astigmatism).

Grade of stereopsis	(Distance) cyl 90° +1		(Distance) cyl 90° +2		(Distance) cyl 90° +3		(Distance) cyl 90° +4	
	*n*	%	*n*	%	*n*	%	*n*	%
Absence	3	7.5	15	37.5	35	87.5	39	97.5
Poor	4	10.0	12	30.0	3	7.5	1	2.5
Moderate	14	35.0	8	20.0	1	2.5	0	0.0
Good	19	47.5	5	12.5	1	2.5	0	0.0
Total	40	100.0	40	100.0	40	100.0	40	100.0

**Table 11:** Results of the post hoc ANOVA test showing a statistical difference between all possible pairs of measurements. Absolute differences between mean rank values are significant if larger than 0.761599273516645 at a confidence level = 0.05.

	(Distance) cyl 90° +1	(Distance) cyl 90° +2	(Distance) cyl 90° +3	(Distance) cyl 90° +4
(Distance) cyl 90° +1	—	1.05	1.925	2.075
(Distance) cyl 90° +2	1.05	—	0.875	1.025
(Distance) cyl 90° +3	1.925	0.875	—	0.15
(Distance) cyl 90° +4	2.075	1.025	0.15	—

**Table 12:** Distribution of patients with different degrees of stereopsis at distance according to the amount of astigmatic anisometropia (“against the rule” astigmatism).

Grade of stereopsis	(Distance) cyl 180° +1		(Distance) cyl 180° +2		(Distance) cyl 180° +3		(Distance) cyl 180° +4	
	*n*	%	*n*	%	*n*	%	*n*	%
Absence	3	7.5	23	57.5	34	85.0	37	92.5
Poor	6	15.0	7	17.5	3	7.5	3	7.5
Moderate	5	12.5	3	7.5	3	7.5	0	0.0
Good	26	65.0	7	17.5	0	0.0	0	0.0
Ogółem	40	100.0	40	100.0	40	100.0	40	100.0

**Table 13:** Results of the post hoc ANOVA test showing a statistical difference between all possible pairs of measurements. Absolute differences between mean rank values are significant if larger than 0.761599273516645 at a confidence level = 0.05.

	(Distance) cyl 180° +1	(Distance) cyl 180° +2	(Distance) cyl 180° +3	(Distance) cyl 180° +4
(Distance) cyl 180° +1	—	1.175	1.8625	2.0125
(Distance) cyl 180° +2	1.175	—	0.6875	0.8375
(Distance) cyl 180° +3	1.8625	0.6875	—	0.15
(Distance) cyl 180° +4	2.0125	0.8375	0.15	—

**Table 14:** Threshold values of myopic anisometropia causing a loss of stereopsis in more than 50% of subjects.

Type of myopic anisometropia	Value in D for near	Value in D for distance
Spherical	3	2
Astigmatism “with the rule”	4	3
Astigmatism “against the rule”	3	2

## Data Availability

The electronic data used to support the findings of this study are available from the corresponding author upon request.
